# Dietary intake of one-carbon nutrients and colorectal cancer risk according to TP53 status

**DOI:** 10.1093/jncics/pkag009

**Published:** 2026-01-29

**Authors:** Shiori Nakano, Taiki Yamaji, Akihisa Hidaka, Taichi Shimazu, Kouya Shiraishi, Aya Kuchiba, Masahiro Saito, Fumihito Kunishima, Ryouji Nakaza, Takashi Kohno, Norie Sawada, Manami Inoue, Shoichiro Tsugane, Motoki Iwasaki

**Affiliations:** Division of Epidemiology, National Cancer Center Institute for Cancer Control, Tokyo, Japan; Division of Epidemiology, National Cancer Center Institute for Cancer Control, Tokyo, Japan; Division of Epidemiology, National Cancer Center Institute for Cancer Control, Tokyo, Japan; Department of Diabetes and Endocrinology, JCHO Tokyo Yamate Medical Centre, Tokyo, Japan; Division of Behavioral Sciences, National Cancer Center Institute for Cancer Control, Tokyo, Japan; Division of Genome Biology, National Cancer Center Research Institute, Tokyo, Japan; Teikyo University Graduate School of Public Health, Tokyo, Japan; Division of Biostatistical Research, Institute for Cancer Control/Biostatistics Division, Center for Research Administration and Support, National Cancer Center, Tokyo, Japan; Department of Diagnostic Pathology, Hiraka General Hospital, Yokote, Japan; Department of Diagnostic Pathology, Okinawa Prefecture Chubu Hospital, Okinawa, Japan; Department of Clinical Laboratory, Nakagami Hospital, Okinawa, Japan; Division of Genome Biology, National Cancer Center Research Institute, Tokyo, Japan; Division of Cohort Research, National Cancer Center Institute for Cancer Control, Tokyo, Japan; Division of Cohort Research, National Cancer Center Institute for Cancer Control, Tokyo, Japan; Division of Prevention, National Cancer Center Institute for Cancer Control, Tokyo, Japan; Division of Cohort Research, National Cancer Center Institute for Cancer Control, Tokyo, Japan; International University of Health and Welfare Graduate School of Public Health, Tokyo, Japan; Division of Epidemiology, National Cancer Center Institute for Cancer Control, Tokyo, Japan; Division of Cohort Research, National Cancer Center Institute for Cancer Control, Tokyo, Japan

## Abstract

**Background:** Accumulating evidence suggests that one-carbon nutrient intake reduces the risk of colorectal cancer (CRC), although folate fortification has been associated with a temporary increase in CRC incidence. We hypothesized that one-carbon nutrients might harbor preventive and protumor effects on CRC according to tumor conditions and investigated whether the effect of one-carbon nutrients on CRC risk differs by TP53 status.

**Methods:** In this prospective study of 21 708 Japanese participants, we applied a multivariable Cox proportional hazards model and examined the associations of dietary intakes of folate, vitamin B6, vitamin B12, and methionine with TP53-overexpressing (*n* = 192), TP53-nonoverexpressing (*n* = 301), *TP53*-mutated (*n* = 180), and *TP53* wild-type (*n* = 134) CRC risk defined by TP53 immunohistochemistry and target sequence.

**Results:** Vitamin B12 and methionine intakes were not associated with any CRC subtypes defined by TP53 status. Meanwhile, folate intake was marginally associated with decreased *TP53*-mutated CRC risk (hazard ratio [HR] with 95% confidence interval [CI] for the highest folate intake quartile compared with the lowest (HR = 0.82, 95% CI = 0.46 to 1.45) and increased *TP53* wild-type CRC risk (HR = 1.50, 95% CI = 0.78 to 2.90). A heterogeneous effect of folate on CRC subtypes was detected (*P* = .03 for heterogeneity between *TP53* mutation statuses). In women, the association between vitamin B6 and CRC also differed by *TP53* mutation status (*P* = .007 for heterogeneity). The hazard ratio of vitamin B6 was 0.71 (95% CI = 0.30 to 1.67) for *TP53*-mutated CRC and 3.89 (95% CI = 1.79 to 8.49) for *TP53* wild-type CRC. However, no heterogeneous effects were observed between TP53 expression statuses.

**Conclusion:** This study supports the hypothesis that the effect of one-carbon nutrient intake on CRC differs according to tumor conditions.

## Introduction

One-carbon metabolism plays an essential role in DNA synthesis and methylation through the transfer of one-carbon units into folate and methionine cycles. Deficiency of the nutrients involved in this metabolism, such as folate, methionine, vitamin B6, and vitamin B12, leads to abnormal one-carbon metabolism. Laboratory studies have revealed that folate, vitamin B6, and vitamin B12 depletion causes DNA damage and/or DNA methylation disruption,^1-4^ resulting in colorectal carcinogenesis in rodent models.^5-7^ Meta-analyses including a large number of participants also suggested that dietary intakes of folate,^8^ methionine,^9^ vitamin B6,^10^ and vitamin B12^11^ were inversely associated with colorectal cancer (CRC) risk. However, the overall effect of one-carbon nutrients on CRC risk remains controversial.^12^

Folic acid fortification in the United States and Canada was associated not with a decrease in CRC incidence but rather temporarily with an increase.^13^ Similarly, a randomized controlled study revealed that folic acid increased the risk of recurrence of advanced and multiple colorectal adenomas, the precursors of most CRC cases.^14^ Some animal studies have also suggested that folate intake promotes the proliferation of precancerous lesions and cancer cells.^6^^,^^15^^,^^16^ These findings imply that one-carbon nutrients may harbor both preventive and protumor effects on CRC, depending on the tumor conditions. Further epidemiological studies considering tumor characteristics are warranted to elucidate the complex role of one-carbon metabolism in colorectal carcinogenesis.

CRC acquires multistep mutations in genes that contribute to genomic stability during its development.^17^ One of the key events in this step is the mutation of *TP53*, which encodes the tumor suppressor protein TP53. *TP53* mutations generally occur at a later stage of CRC development^17^^,^^18^ and are rare in adenomas but are found in approximately half of CRC cases.^19^ In clinical settings, TP53 expression detected by immunohistochemistry is commonly used as a surrogate marker for *TP53* mutation status, as *TP53* missense mutations often result in the accumulation of aberrant TP53.

Notably, *TP53* strand breaks and TP53 overexpression have been observed during folate depletion.^20-22^ This suggests that one-carbon nutrients may help prevent *TP53-*mutated or TP53-overexpressing CRC. In contrast, CRC with *TP53* wild-type seems to behave like adenomas in which *TP53* is infrequently mutated. In such cases, one-carbon nutrients could promote tumor growth. Investigating the association between one-carbon nutrient intake and CRC risk according to TP53 status may provide important clues regarding the etiology of one-carbon metabolism and CRC risk.

Few prospective studies have examined whether the effect of one-carbon nutrient intake on CRC risk differs according to TP53 status, and their results have been inconsistent.^23^^,^^24^ These studies assessed TP53 status based solely on protein expression using immunohistochemistry.^23^^,^^24^ However, TP53 overexpression does not capture all *TP53* mutations, as *TP53* nonsense mutations, although relatively infrequent among *TP53* mutations, typically result in the loss of TP53 protein expression.^25-27^ Therefore, the absence of TP53-overexpression does not necessarily indicate the presence of wild-type *TP53*. Although TP53 expression status may reflect tumor phenotype and clinical behavior better than *TP53* mutation status, there is currently no consensus on whether protein expression status or mutation status is the optimal method for defining TP53 status.

Here, we evaluated TP53 expression status using immunohistochemistry and *TP53* mutation status using DNA sequencing in CRC. We then investigated the effect of one-carbon nutrient intake on CRC risk according to both TP53 expression status (overexpressing vs nonoverexpressing) and *TP53* mutation status (mutated vs wild-type), in a Japanese prospective longitudinal study.

## Materials and methods

### Study population

**Figure 1.**
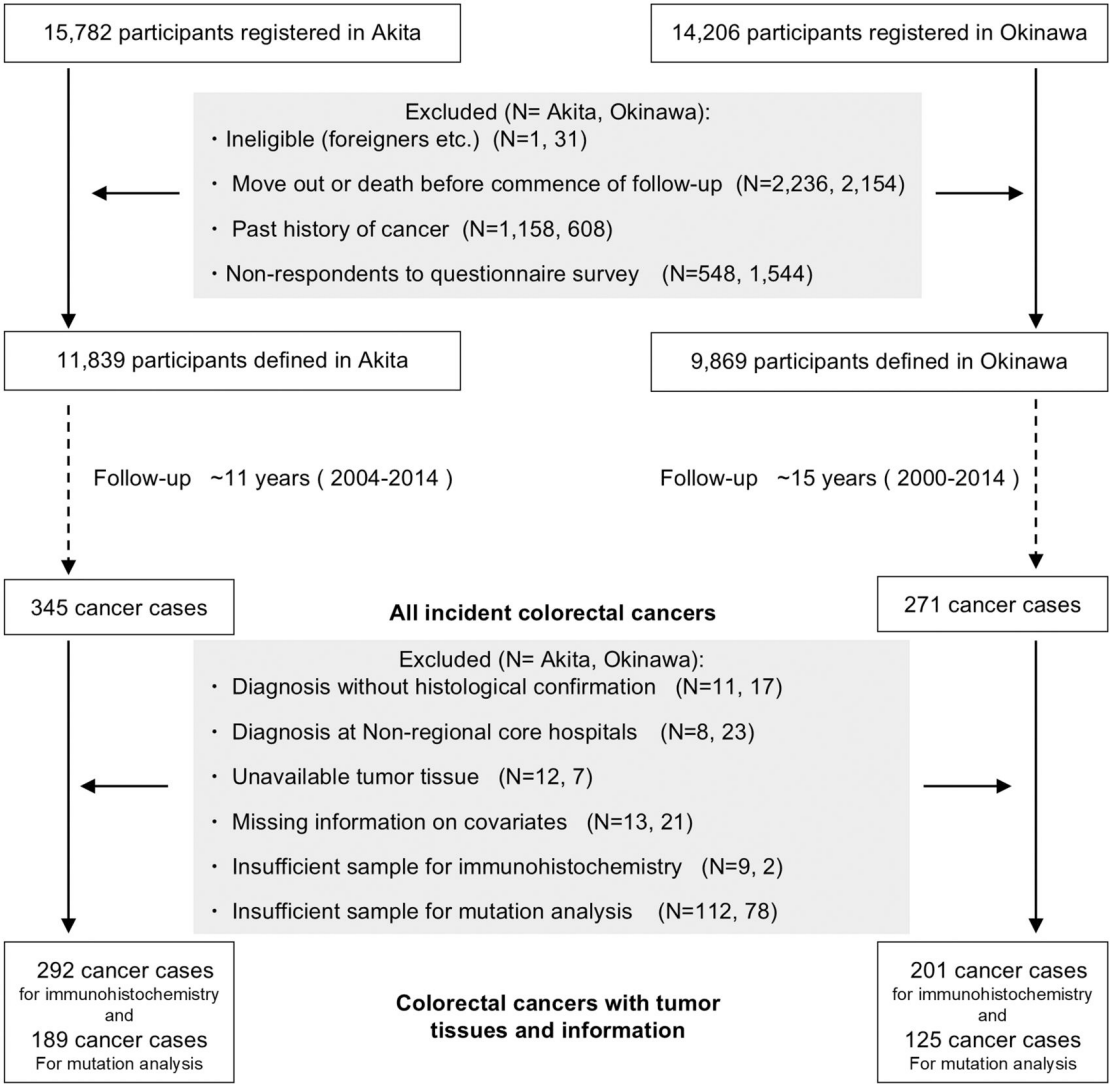
Study flow.

The Japan Public Health Center-based Prospective (JPHC) study enrolled middle-aged and older residents across 11 public health center areas between 1990 and 1994 and conducted follow-up surveys at 5-year intervals to investigate lifestyle factors and common diseases.^28^ Among the 11 areas of the JPHC study, this study included 29 988 participants from the Akita and Okinawa areas of northern and southern Japan, respectively. After excluding participants who were ineligible, were nonresponders to the questionnaire, had a history of cancer, died, or moved out before the initiation of this study corresponding to tumor collection (2004 in Akita and 2000 in Okinawa), 21 708 participants were defined and followed up until December 31, 2014 (see Figure1). Participants were informed of the JPHC study objectives and provided consent to participate in the JPHC study. Before tissue collection, information regarding this study was posted on our center’s website to provide participants with the opportunity to opt out at any time. The comprehensive study protocol, including this study, was approved by the institutional review board of the National Cancer Center, Tokyo, Japan (Approval No. 2014-214).

### Dietary assessment

In this study, one-carbon nutrients refer to dietary components involved in the one-carbon metabolic pathway. Specifically, folate, methionine, vitamin B6, and vitamin B12 intakes were estimated using a food frequency questionnaire administered in 2000 and the Standard Tables of Food Composition in Japan (7th revised edition). The estimated dietary intakes were energy-adjusted using the residual method and subsequently categorized into quartiles or tertiles based on their sex-specific distributions. Other nutrient variables included as covariates were similarly categorized into tertiles (Supplementary Methods).

### CRC ascertainment

Newly diagnosed cancer was mainly ascertained using population-based cancer registries and active patient notifications from regional hospitals. Information on cancer site and histology was coded using the *International Classification of Diseases for Oncology Third Edition* (ICD-O-3), and C18-C20 were allocated to CRC. At the end of the follow-up period, 616 CRC cases were identified. Formalin-fixed, paraffin-embedded tumor tissues were collected from the Department of Pathology at regional core hospitals where they had been stored: Hiraka General Hospital and Yokote Municipal Hospital in Akita and Okinawa Prefecture Chubu Hospital and Nakagami Hospital in Okinawa (see Figure  1). Eventually, 538 CRC tissue samples were collected, yielding a retrieval rate of approximately 90%. The clinical characteristics of the participants with and without tumor tissue were comparable.^29^

### Definition of TP53 expression and *TP53* mutation statuses

Formalin-fixed, paraffin-embedded tumor tissues were used for TP53 immunohistochemistry and target sequencing. Immunohistochemistry for TP53 expression was performed using the Autostainer Link48 (Agilent Technologies, CA, USA) with a mouse monoclonal anti-p53 antibody (DO-7, Agilent Technologies) and the EnVision FLEX/HRP system. Among 538 CRC cases, TP53 expression was successfully assessed in 527 cases. The proportion of tumor cell nuclei showing positive staining was assessed in 10% increments by a trained laboratory technician blinded to mutation and patient data. *TP53* mutation status was determined as previously described.^29^ Briefly, target sequencing was performed using the Ion AmpliSeq Cancer Hotspot Panel v2 on the Ion Proton platform (Thermo Fisher Scientific, MA, USA). Pathogenic or oncogenic *TP53* variants were identified using ANNOVAR^30^ and OncoKB.^31^^,^^32^ After quality control, sequencing data were available for 397 cases, with no substantial differences in clinical characteristics between cases with and without successful sequencing.^33^

### Statistical analysis

Person-years for each participant were calculated from the start of follow-up to any of the following censor points, whichever came first: incident CRC, death, relocation from the study area, or end of follow-up. In this study, the median person-years was 11 years. The associations of one-carbon nutrient intake with overall CRC and subtypes defined by TP53 status were evaluated using hazard ratios (HRs) and 95% confidence intervals (CIs) using two Cox proportional hazards models. For the crude model, the hazard ratio was adjusted for sex and age and stratified by study area, whereas, for the multivariable model, hazard ratio was further adjusted for body mass index, smoking status, physical activity (metabolic equivalent of tasks, hours per day), CRC screening, history of diabetes, vitamin B supplement use, intake of calcium and fiber, and intake of one-carbon nutrients, except for exposure. After confirming that the distributions of exposures and covariates were similar between the 2000 and 1995 surveys, missing information on these variables in the 2000 survey was imputed using data from the 1995 survey. Approximately 6% of participants had missing information in both surveys and were excluded from the analyses. Eventually, this study included 493 cases with TP53 expression levels and 314 cases with *TP53* mutation status.

Using the receiver operating characteristic (ROC) curve and Youden index,^34^ we identified the TP53 expression level that best predicted *TP53* mutation status and determined the optimal cutoff point for classifying TP53 expression status as overexpressing or nonoverexpressing.

The associations were analyzed separately according to TP53 expression and *TP53* mutation status using the duplication method. Heterogeneity in the associations was evaluated using a likelihood ratio test under the null hypothesis that the effects of exposure on CRC are consistent across each status.^35^ For sensitivity analysis, the aforementioned analyses were performed in subgroups defined by sex or drinking status, factors known to influence the absorption and degradation of vitamin B6 and folate. The statistical significance level was set at a 2-sided *P* value less than .05, and all statistical analyses were performed using SAS v9.4 (SAS Institute, NC, USA).

## Results

### Classification of *TP53* mutation and TP53 expression status

Of the 314 participants included for mutation analysis, 129 had nonsynonymous missense mutations and 51 had stop-gain frameshift or nonsense mutations. In 309 cases with information on *TP53* mutation and TP53 expression status, a ROC curve for TP53 overexpression as a surrogate of *TP53* missense mutations was generated with an area under the ROC curve of 0.87 (Figure S1). The optimal cutoff value for TP53 overexpression was determined to be 40% using the maximum Youden index (Table 1) and was applied in 493 cases for immunohistochemical analysis. Finally, 192 cases with TP53 overexpression, 301 with TP53 nonoverexpression, 180 with any *TP53* mutation, and 134 with wild-type *TP53* were identified.

**Table 1.** Prediction values of *TP53* missense mutations using TP53 expression

TP53 positive cells (%)	*TP53* mutation			Sensitivity	Specificity	Youden index
	Missense	Stop-gain	Wild-type			
0	10	32	65	1.000	0.000	0.000
10	12	7	40	0.922	0.536	0.458
20	2	3	8	0.828	0.796	0.624
30	7	2	8	0.813	0.856	0.668
40	3	1	2	0.758	0.912	0.669
50	17	1	2	0.734	0.928	0.663
60	17	1	1	0.602	0.945	0.546
70	23	2	2	0.469	0.956	0.425
80	23	1	1	0.289	0.978	0.267
90	11	0	0	0.109	0.989	0.098
100	3	1	1	0.023	0.989	0.012

### Participant characteristics

Participant characteristics according to folate, vitamin B6, vitamin B12, and methionine intake are shown in Table 2 and Supplementary Table S1. Regarding alcohol consumption, while participants with high folate and methionine intakes consumed fewer alcoholic beverages, those with high vitamin B6 and vitamin B12 intakes consumed more compared with participants with low nutrient intakes.

**Table 2.** Baseline characteristics of participants according to dietary intake of folate and vitamin B6

Characteristics	Folate				Vitamin B6			
	Q1 (*n* = 4994)	Q2 (*n* = 5079)	Q3 (*n* = 5087)	Q4 (*n* = 5094)	Q1 (*n* = 5031)	Q2 (*n* = 5062)	Q3 (*n* = 5077)	Q4 (*n* = 5084)
Age, mean (SD), y	60.0 (6.2)	61.2 (6.2)	61.9 (6.2)	62.7 (6.2)	59.6 (6.1)	61.1 (6.2)	62.1 (6.2)	63.0 (6.1)
Men, No. (%)	2331 (46.7)	2371 (46.7)	2361 (46.4)	2360 (46.3)	2351 (46.7)	2359 (46.6)	2357 (46.4)	2356 (46.3)
Body mass index, mean (SD), kg/m^2^	24.1 (3.3)	23.9 (3.1)	23.9 (3.1)	24.0 (3.1)	24.1 (3.3)	24.0 (3.2)	23.9 (3.1)	23.9 (3.1)
Metabolic equivalent for tasks, mean (SD), h/d	33.4 (5.8)	33.5 (5.7)	33.6 (5.7)	33.2 (5.6)	33.5 (5.8)	33.5 (5.6)	33.4 (5.7)	33.4 (5.7)
Diabetes, No. (%)	224 (4.5)	250 (4.9)	267 (5.2)	342 (6.7)	226 (4.5)	213 (4.2)	284 (5.6)	360 (7.1)
Colorectal screening, No. (%)	354 (7.1)	490 (9.6)	537 (10.6)	577 (11.3)	360 (7.2)	464 (9.2)	512 (10.1)	622 (12.2)
Smoking, No. (%)								
Never	3090 (61.9)	3305 (65.1)	3425 (67.3)	3444 (67.6)	3098 (61.6)	3307 (65.3)	3416 (67.3)	3443 (67.7)
Past	614 (12.3)	663 (13.1)	661 (13.0)	708 (13.9)	596 (11.8)	660 (13.0)	666 (13.1)	724 (14.2)
Current	1290 (25.8)	1111 (21.9)	1001 (19.7)	942 (18.5)	1337 (26.6)	1095 (21.6)	995 (19.6)	917 (18.0)
Alcohol drinking, No. (%)								
Non	2972 (59.5)	3053 (60.1)	3082 (60.6)	3340 (65.6)	3246 (64.5)	3230 (63.8)	3061 (60.3)	2910 (57.2)
Occasional	481 (9.6)	616 (12.1)	682 (13.4)	717 (14.1)	464 (9.2)	582 (11.5)	753 (14.8)	697 (13.7)
Light	455 (9.1)	497 (9.8)	559 (11.0)	505 (9.9)	409 (8.1)	500 (9.9)	517 (10.2)	590 (11.6)
Heavy	1086 (21.7)	913 (18.0)	764 (15.0)	532 (10.4)	912 (18.1)	750 (14.8)	746 (14.7)	887 (17.4)
Vitamin B supplement use, No. (%)	482 (9.7)	608 (12.0)	612 (12.0)	590 (11.6)	469 (9.3)	563 (11.1)	599 (11.8)	661 (13.0)
Dietary intake, median (IQR)^a^								
Energy, kcal/d	1857.6 (1423.9-2388.7)	1898.8 (1507.3-2398.1)	1906.0 (1521.3-2395)	1819.2 (1443.7-2286.5)	1926.8 (1538.9-2397.5)	1786.1 (1416-2253.1)	1809.4 (1443.9-2309.5)	1964.9 (1507.5-2532.6)
Calcium, mg/d	343.6 (249.3-476.9)	430.1 (337.3-553.2)	493.8 (398.8-616.2)	587.9 (478.9-721)	373.6 (269.2-509.8)	446.7 (340.3-577.8)	495.8 (389.8-628.6)	556.0 (443.7-698)
Fiber, g/d	9.7 (7.8-11.5)	12.9 (10.9-15)	15.3 (13-17.7)	19.3 (16.1-22.7)	10.6 (8.4-13)	13.1 (10.6-15.7)	15.2 (12.2-18.2)	17.9 (14.3-21.9)
Folate, g/d	238.9 (201-265.3)	334.0 (312.1-355.9)	422.4 (399-450)	564.5 (516.2-646.2)	272.6 (216.8-333.7)	354.3 (288.7-424.2)	415.6 (343.6-496.1)	498.2 (404.2-609.3)
Vitamin B6, mg/d	2.2 (2.1-2.4)	2.4 (2.2-2.6)	2.5 (2.4-2.7)	2.7 (2.5-2.9)	2.1 (2-2.2)	2.4 (2.3-2.4)	2.6 (2.5-2.6)	2.8 (2.8-3)
Vitamin B12, g/d	6.0 (4.3-8.4)	7.6 (5.4-10.4)	8.3 (5.8-11.3)	8.4 (5.7-11.9)	4.9 (3.7-6.5)	6.8 (5.1-8.9)	8.6 (6.4-11.1)	11.1 (8.2-14.4)
Methionine, mg/d	1340.7 (1153.6-1545.9)	1426.4 (1248.6-1639.8)	1458.6 (1275.2-1668.6)	1467.7 (1268.7-1698.8)	1242.1 (1092.5-1385.7)	1384.5 (1231.3-1540.7)	1492.0 (1316.9-1666.9)	1657.1 (1438.8-1885)

Abbreviation: IQR = interquartile range.

a The intake was adjusted for energy intake using a residual method.

Characteristics according to TP53 status are summarized in Table 3. Although the clinical characteristics of CRC cases were generally comparable across TP53 statuses, individuals with TP53-nonoverexpressing or *TP53* wild-type CRC consumed more energy than those with TP53-overexpressing or *TP53*-mutated CRC. Moreover, *TP53* wild-type CRC cases showed a higher prevalence of heavy drinking compared with other CRC groups.

**Table 3.** Clinical characteristics of participants according to TP53 status

TP53 status	Immunohistochemistry		DNA sequence	
	Overexpression (*n* = 192)	Nonoverexpression (*n* = 301)	Mutation (*n* = 180)	Wild-type (*n* = 134)
Men, No. (%)	120 (62.5)	181 (60.1)	112 (62.2)	87 (64.9)
Age at diagnosis, mean (SD)	70.0 (6.5)	69.4 (6.1)	70.5 (6.1)	69.4 (6.5)
Sites, No. (%)				
Rectal	51 (26.6)	70 (23.3)	42 (23.3)	32 (23.9)
Colon	141 (73.4)	231 (76.7)	138 (76.7)	102 (76.1)
Proximal	79 (41.1)	145 (48.2)	70 (38.9)	64 (47.8)
Distal	57 (29.7)	83 (27.6)	63 (35.0)	36 (26.9)
Differentiation, No. (%)				
High	70 (36.5)	130 (43.2)	72 (40.0)	49 (36.6)
Middle	76 (39.6)	97 (32.2)	63 (35.0)	40 (29.9)
Low	4 (2.1)	7 (2.3)	1 (0.6)	4 (3.0)
Stage, No. (%)				
Localized	107 (55.7)	192 (63.8)	114 (63.3)	86 (64.2)
Regional	55 (28.6)	67 (22.3)	39 (21.7)	32 (23.9)
Distant	26 (13.5)	37 (12.3)	22 (12.2)	14 (10.4)
Body mass index, mean (SD), kg/m^2^	24.0 (3.2)	24.1 (3.4)	24.2 (3.5)	24.0 (3.5)
Metabolic equivalent for tasks, mean (SD), h/d	32.4 (5.3)	33.2 (5.7)	32.7 (5.7)	33.8 (5.7)
Diabetes, No. (%)	15 (7.8)	18 (6.0)	18 (10.0)	6 (4.5)
Colorectal screening, No. (%)	8 (4.2)	17 (5.6)	6 (3.3)	8 (6.0)
Smoking, No. (%)				
Never	103 (53.6)	158 (52.5)	94 (52.2)	65 (48.5)
Past	35 (18.2)	65 (21.6)	34 (18.9)	33 (24.6)
Current	54 (28.1)	78 (25.9)	52 (28.9)	36 (26.9)
Alcohol drinking, No. (%)				
Non or occasional	89 (46.4)	159 (52.8)	92 (51.1)	55 (41.0)
Light	35 (18.2)	29 (9.6)	31 (17.2)	11 (8.2)
Heavy	68 (35.4)	113 (37.5)	57 (31.7)	68 (50.7)
Vitamin B supplement use, No. (%)	16 (8.3)	35 (11.6)	17 (9.4)	12 (9.0)
Dietary intake, median (IQR)^a^				
Energy, kcal/d	1811.8 (1489.8-2274.2)	2000.4 (1576.1-2439.2)	1852.9 (1486.3-2320.7)	2077.3 (1661.1-2473)
Calcium, mg/d	464.7 (333.2-666.6)	455.6 (351.6-565.4)	476 (334.1-644.6)	443.4 (356.6-584.7)
Fiber, g/d	13.4 (10.7-17.3)	13.8 (10.7-17.4)	13.5 (10.5-17.5)	13.8 (10.8-17.8)
Folate, g/d	375 (290.4-469)	375.6 (296.4-483.2)	360.5 (295.2-467.2)	384 (296.2-498.5)
Vitamin B6, mg/d	2.5 (2.3-2.7)	2.5 (2.3-2.7)	2.5 (2.3-2.7)	2.6 (2.3-2.8)
Vitamin B12, g/d	8.1 (5.6-11.1)	7.4 (5.2-10.7)	7.6 (5.2-10.4)	8.3 (5.5-13)
Methionine, mg/d	1468.9 (1256.9-1740.1)	1423.1 (1207.1-1664)	1477.2 (1252.4-1689.5)	1407.2 (1194.8-1707.4)

Abbreviation: IQR = interquartile range.

a The intake was adjusted for energy intake using a residual method.

### Association between intake of one-carbon nutrients and CRC

One-carbon nutrient intake was not associated with overall CRC in the crude (Table S2) or multivariable model (Table 4). Additionally, no associations or heterogeneities were found in CRC defined by TP53 expression status (Table S2 and Table 4). In contrast to TP53 expression-defined CRC, heterogeneous effects of folate on CRC subtypes classified by *TP53* mutation status were detected (*P* = .03 for heterogeneity; Table 4). The multivariable hazard ratios for the highest folate intake quartile compared with the lowest were 0.82 (95% CI = 0.46 to 1.45) for *TP53*-mutated CRC and 1.50 (95% CI = 0.78 to 2.90) for *TP53* wild-type CRC. No heterogeneous effects of vitamin B6 on CRC subtypes classified by *TP53* mutation status were detected, whereas vitamin B6 was associated with increased risk of *TP53* wild-type CRC (HR = 1.94, 95% CI = 1.03 to 3.66). The positive association of vitamin B6 intake with *TP53* wild-type CRC persisted in women but not in men (Table 5). The association between vitamin B6 intake and CRC in women differed by *TP53* mutation status (*P* = .007 for heterogeneity). The multivariable hazard ratios for the highest vitamin B6 intake tertile compared with the lowest in women were 0.71 (95% CI = 0.30 to 1.67) for *TP53*-mutated CRC and 3.89 (95% CI = 1.79 to 8.49) for *TP53* wild-type CRC. In the sensitivity analysis stratified by drinking status (Table 6), albeit with no statistical significance, the positive estimation for the associations of folate and vitamin B6 intakes with *TP53* wild-type CRC remained in non- or occasional drinkers. Meanwhile, in regular drinkers, the positive associations with *TP53* wild-type CRC disappeared, and the inverse associations of vitamin B6 intake with overall and TP53-overexpressing CRC were observed (HR = 0.41, 95% CI = 0.22 to 0.75, for overall CRC, and HR = 0.31, 95% CI = 0.11 to 0.84, for TP53-overexpressing CRC).

**Table 4.** Associations of one-carbon nutrients with colorectal cancer defined by TP53 status

Nutrients	Overall		Immunohistochemistry				DNA sequence			
			Overexpression		Nonoverexpression		Mutation		Wild-type	
	No.	HR^a^ (95% CI)	No.	HR^a^ (95% CI)	No.	HR^a^ (95% CI)	No.	HR^a^ (95% CI)	No.	HR^a^ (95% CI)
Folate^d^										
Q1	113	1 (referent)	43	1 (referent)	68	1 (referent)	39	1 (referent)	30	1 (referent)
Q2	129	1.08 (0.82 to 1.42)	52	1.12 (0.73 to 1.72)	74	1.03 (0.72 to 1.48)	56	1.33 (0.85 to 2.08)	26	0.87 (0.5 to 1.52)
Q3	135	1.08 (0.8 to 1.47)	50	1.01 (0.63 to 1.63)	82	1.09 (0.72 to 1.63)	44	0.97 (0.59 to 1.59)	39	1.34 (0.74 to 2.43)
Q4	127	1.00 (0.7 to 1.42)	47	0.91 (0.51 to 1.63)	77	1.00 (0.63 to 1.57)	41	0.82 (0.46 to 1.45)	39	1.50 (0.78 to 2.9)
*P*_trend_		.99		.68		.96		.26		.13
*P*_heterogeneity_						.93^b^				.03^c^
Vitamin B6^e^										
Q1	110	1 (referent)	36	1 (referent)	73	1 (referent)	37	1 (referent)	24	1 (referent)
Q2	114	0.98 (0.74 to 1.3)	47	1.24 (0.77 to 1.97)	62	0.8 (0.55 to 1.14)	46	1.29 (0.81 to 2.06)	31	1.23 (0.7 to 2.16)
Q3	142	1.21 (0.9 to 1.62)	56	1.41 (0.86 to 2.3)	84	1.07 (0.74 to 1.56)	53	1.53 (0.95 to 2.48)	30	1.19 (0.64 to 2.21)
Q4	138	1.14 (0.81 to 1.62)	53	1.33 (0.73 to 2.42)	82	1.01 (0.66 to 1.56)	44	1.34 (0.73 to 2.46)	49	1.94 (1.03 to 3.66)
*P*_trend_		.27		.35		.59		.29		.04
*P*_heterogeneity_						.53^b^				.29^c^
Vitamin B12^f^										
Q1	115	1 (referent)	41	1 (referent)	72	1 (referent)	45	1 (referent)	29	1 (referent)
Q2	121	1.01 (0.77 to 1.31)	39	0.93 (0.59 to 1.48)	79	1.02 (0.73 to 1.43)	44	0.93 (0.6 to 1.44)	29	0.93 (0.55 to 1.58)
Q3	130	0.99 (0.73 to 1.35)	57	1.30 (0.78 to 2.15)	70	0.81 (0.55 to 1.2)	47	0.91 (0.54 to 1.54)	27	0.81 (0.45 to 1.47)
Q4	138	0.97 (0.68 to 1.37)	55	1.17 (0.65 to 2.1)	80	0.84 (0.54 to 1.3)	44	0.78 (0.42 to 1.41)	49	1.50 (0.82 to 2.75)
*P*_trend_		.88		.42		.31		.44		.23
*P*_heterogeneity_						.25^b^				.11^c^
Methionine^g^										
Q1	135	1 (referent)	45	1 (referent)	85	1 (referent)	45	1 (referent)	42	1 (referent)
Q2	111	0.79 (0.6 to 1.04)	42	0.84 (0.53 to 1.32)	66	0.78 (0.55 to 1.1)	37	0.79 (0.5 to 1.26)	32	0.72 (0.44 to 1.19)
Q3	112	0.81 (0.6 to 1.09)	42	0.80 (0.47 to 1.34)	69	0.86 (0.59 to 1.25)	44	0.97 (0.59 to 1.58)	20	0.44 (0.24 to 0.8)
Q4	146	1.01 (0.72 to 1.4)	63	1.09 (0.62 to 1.91)	81	1.00 (0.66 to 1.52)	54	1.16 (0.64 to 2.1)	40	0.84 (0.49 to 1.44)
*P*_trend_		.86		.69		.90		.50		.43
*P*_heterogeneity_						.93^b^				.19^c^

Abbreviations: HR = hazard ratio; CI = confidence interval.

a Hazard ratio was stratified by study area and adjusted for age, sex, smoking status, body mass index, physical activity, diabetes, dietary intake of calcium and fiber, colorectal cancer screening, vitamin B supplement use, alcohol, and one-carbon nutrients except for exposure.

b *P* value for heterogeneity between TP53 expression status.

c *P* value for heterogeneity between *TP53* mutation status.

d Quartiles of folate (g/d) were <271.2, 271.3-355.0, 355.0-457.5, and ≥457.5 for men and <309.2, 309.3-395.6, 395.7-497.9, and ≥497.9 for women.

e Quartiles of vitamin B6 (mg/d) were <2.30, 2.30-2.51, 2.51-2.74, and ≥2.74 for men and <2.24, 2.24-2.42, 2.42-2.62, and ≥2.62 for women.

f Quartiles of vitamin B12 (g/d) were <5.12, 5.12-7.44, 7.44-10.50, and ≥10.50 for men and <5.17, 5.17-7.56, 7.56-10.58, and ≥10.58 for women.

g Quartiles of methionine (mg/d) were <1259.1, 1259.3-1475.4, 1475.4-1713.7, and ≥1713.8 for men and <1216.5, 1216.7-1389.0, 1389.0-1583.8, and >1583.9 for women.

**Table 5.** Hazard ratios^a^ of TP53-defined colorectal cancer subtypes according to one–carbon nutrients by sex

Nutrients	Men					Women				
Types	T1	T2	T3	*P*_trend_	*P*_heterogeneity_	T1	T2	T3	*P*_trend_	*P*_heterogeneity_
Folate^d^										
Overall, No.	93	108	108			61	73	61		
	1 (referent)	1.09 (0.8 to 1.49)	1.09 (0.75 to 1.58)	.66		1 (referent)	1.05 (0.7 to 1.59)	0.85 (0.51 to 1.42)	.52	
Overexpression	36	38	46			22	29	21		
	1 (referent)	1.06 (0.65 to 1.7)	1.40 (0.8 to 2.43)	.23		1 (referent)	1.03 (0.5 to 2.11)	0.71 (0.29 to 1.72)	.42	
Nonoverexpression	54	69	58			38	42	40		
	1 (referent)	1.11 (0.73 to 1.67)	0.86 (0.52 to 1.42)	.52	.22^b^	1 (referent)	1.02 (0.61 to 1.72)	0.95 (0.5 to 1.8)	.88	.74^b^
Mutation	37	36	39			21	27	20		
	1 (referent)	0.79 (0.48 to 1.31)	0.74 (0.39 to 1.38)	.37		1 (referent)	1.31 (0.66 to 2.6)	1.06 (0.46 to 2.46)	.87	
Wild-type	26	33	28			12	15	20		
	1 (referent)	1.21 (0.67 to 2.2)	1.10 (0.55 to 2.21)	.79	.55^c^	1 (referent)	1.17 (0.48 to 2.83)	1.58 (0.53 to 4.64)	.39	.57^c^
Vitamin B6^e^										
Overall	98	111	100			58	60	77		
	1 (referent)	1.02 (0.76 to 1.37)	0.86 (0.59 to 1.27)	.45		1 (referent)	0.89 (0.58 to 1.36)	1.06 (0.64 to 1.75)	.72	
Overexpression	30	51	39			23	23	26		
	1 (referent)	1.62 (0.99 to 2.66)	1.23 (0.63 to 2.42)	.62		1 (referent)	0.65 (0.31 to 1.34)	0.57 (0.24 to 1.37)	.24	
Nonoverexpression	65	59	57			33	37	50		
	1 (referent)	0.77 (0.53 to 1.13)	0.68 (0.42 to 1.09)	.12	.07^b^	1 (referent)	1.13 (0.67 to 1.92)	1.61 (0.89 to 2.92)	.10	.15^b^
Mutation	29	47	36			24	23	21		
	1 (referent)	1.70 (1.05 to 2.74)	1.39 (0.73 to 2.67)	.34		1 (referent)	0.83 (0.4 to 1.71)	0.71 (0.3 to 1.67)	.44	
Wild-type	28	27	32			9	13	25		
	1 (referent)	0.77 (0.43 to 1.38)	0.76 (0.37 to 1.56)	.48	.12^c^	1 (referent)	1.68 (0.68 to 4.1)	3.89 (1.79 to 8.49)	.0002	.007^c^
Vitamin B12^f^										
Overall	97	101	111			59	61	75		
	1 (referent)	1.06 (0.78 to 1.45)	1.10 (0.75 to 1.62)	.60		1 (referent)	0.81 (0.53 to 1.23)	0.80 (0.48 to 1.31)	.41	
Overexpression	34	42	44			20	22	30		
	1 (referent)	1.30 (0.77 to 2.2)	1.28 (0.64 to 2.54)	.48		1 (referent)	0.81 (0.39 to 1.65)	0.91 (0.39 to 2.14)	.90	
Nonoverexpression	61	57	63			38	38	44		
	1 (referent)	0.92 (0.63 to 1.36)	0.96 (0.61 to 1.53)	.88	.58^b^	1 (referent)	0.79 (0.47 to 1.32)	0.71 (0.38 to 1.3)	.28	.82^b^
Mutation	33	44	35			23	23	22		
	1 (referent)	1.29 (0.77 to 2.16)	0.90 (0.45 to 1.81)	.75		1 (referent)	0.86 (0.43 to 1.73)	0.75 (0.32 to 1.76)	.52	
Wild-type	27	27	33			14	10	23		
	1 (referent)	0.87 (0.5 to 1.51)	0.90 (0.51 to 1.59)	.73	.44^c^	1 (referent)	0.59 (0.24 to 1.44)	1.32 (0.49 to 3.59)	.48	.19^c^
Methionine^g^										
Overall	115	98	96			58	60	77		
	1 (referent)	0.80 (0.58 to 1.1)	0.75 (0.51 to 1.08)	.63		1 (referent)	0.94 (0.64 to 1.38)	1.15 (0.74 to 1.78)	.15	
Overexpression	41	37	42			19	19	34		
	1 (referent)	0.80 (0.47 to 1.37)	0.77 (0.42 to 1.41)	.13		1 (referent)	0.81 (0.4 to 1.63)	1.46 (0.66 to 3.23)	.50	
Nonoverexpression	69	60	52			37	40	43		
	1 (referent)	0.85 (0.56 to 1.26)	0.75 (0.46 to 1.22)	.33	.96^b^	1 (referent)	1.05 (0.66 to 1.66)	1.04 (0.61 to 1.77)	.52	.31^b^
Mutation	37	35	40			20	20	28		
	1 (referent)	0.85 (0.5 to 1.44)	0.85 (0.45 to 1.6)	.26		1 (referent)	1.04 (0.54 to 2)	1.79 (0.8 to 3.97)	.88	
Wild-type	35	28	24			16	12	19		
	1 (referent)	0.80 (0.46 to 1.41)	0.72 (0.38 to 1.37)	.43	.93^c^	1 (referent)	0.58 (0.26 to 1.25)	0.73 (0.35 to 1.52)	.27	.25^c^

a Hazard ratio was stratified by study area and adjusted for age, smoking status, body mass index, physical activity, diabetes, dietary intake of calcium and fiber, colorectal cancer screening, vitamin B supplement use, alcohol, and one-carbon nutrients except for exposure.

b *P* value for heterogeneity between TP53 expression status.

c *P* value for heterogeneity between *TP53* mutation status.

d Tertiles of folate (g/d) were <298.8, 298.8-416.8, and ≥416.8 for men and <338.6, 338.6-458.9, and ≥458.9 for women.

e Tertiles of vitamin B6 (mg/d) were <2.37, 2.37-2.65, and ≥2.65 for men and <2.30, 2.30-2.55, and ≥2.55 for women.

f Tertiles of vitamin B12 (g/d) were <5.83, 5.83-9.28, and ≥9.28 for men and <5.97, 5.97-9.37, and ≥9.37 for women.

g Tertiles of methionine (mg/d) were <1334.7, 1334.8-1624.4, and ≥1624.4 for men and <1277.4, 1277.4-1507.1, and ≥1507.3 for women.

**Table 6.** Hazard ratios^a^ of TP53-defined colorectal cancer subtypes according to one-carbon nutrients by alcohol intake

Nutrients	Non- or occasional drinkers					Regular drinkers				
Types	T1	T2	T3	*P*_trend_	*P*_heterogeneity_	T1	T2	T3	*P*_trend_	*P*_heterogeneity_
Folate^d^										
Overall	76	88	87			78	93	82		
	1 (referent)	0.98 (0.65 to 1.47)	0.84 (0.47 to 1.49)	.55		1 (referent)	1.19 (0.81 to 1.76)	1.17 (0.67 to 2.04)	.59	
Overexpression	27	32	30			31	35	37		
	1 (referent)	1.16 (0.6 to 2.22)	1.12 (0.42 to 2.93)	.82		1 (referent)	1.23 (0.68 to 2.23)	1.64 (0.66 to 4.05)	.28	
Nonoverexpression	48	54	57			44	57	41		
	1 (referent)	0.89 (0.53 to 1.49)	0.76 (0.37 to 1.58)	.47	.68^b^	1 (referent)	1.17 (0.69 to 1.99)	0.88 (0.42 to 1.82)	.66	.64^b^
Mutation	27	33	32			31	30	27		
	1 (referent)	1.28 (0.67 to 2.43)	1.25 (0.47 to 3.29)	.68		1 (referent)	0.98 (0.52 to 1.87)	1.27 (0.44 to 3.64)	.73	
Wild-type	13	15	27			25	33	21		
	1 (referent)	0.97 (0.36 to 2.61)	1.57 (0.39 to 6.25)	.43	.60^c^	1 (referent)	0.90 (0.41 to 1.96)	0.55 (0.2 to 1.54)	.20	.17^c^
Vitamin B6^e^										
Overall	75	87	89			81	84	88		
	1 (referent)	0.99 (0.65 to 1.5)	1.00 (0.55 to 1.82)	.97		1 (referent)	0.60 (0.38 to 0.95)	0.41 (0.22 to 0.75)	.004	
Overexpression	23	36	30			30	38	35		
	1 (referent)	1.51 (0.74 to 3.08)	1.54 (0.53 to 4.43)	.43		1 (referent)	0.57 (0.26 to 1.22)	0.31 (0.11 to 0.84)	.02	
Nonoverexpression	50	51	58			48	45	49		
	1 (referent)	0.75 (0.43 to 1.3)	0.77 (0.37 to 1.6)	.57	.44^b^	1 (referent)	0.62 (0.35 to 1.11)	0.47 (0.21 to 1.01)	.06	.57^b^
Mutation	29	37	26			24	33	31		
	1 (referent)	1.22 (0.63 to 2.37)	0.99 (0.37 to 2.6)	.99		1 (referent)	0.95 (0.46 to 1.93)	0.65 (0.24 to 1.75)	.33	
Wild-type	13	17	25			24	23	32		
	1 (referent)	1.33 (0.55 to 3.15)	2.26 (0.71 to 7.11)	.13	.19^c^	1 (referent)	0.61 (0.27 to 1.38)	0.59 (0.19 to 1.77)	.39	.34^c^
Vitamin B12^f^										
Overall	78	81	92			78	81	94		
	1 (referent)	1.00 (0.67 to 1.5)	1.07 (0.61 to 1.89)	.78		1 (referent)	0.94 (0.63 to 1.39)	0.94 (0.52 to 1.68)	.85	
Overexpression	25	25	39			29	39	35		
	1 (referent)	0.70 (0.31 to 1.61)	0.87 (0.3 to 2.52)	.98		1 (referent)	1.10 (0.58 to 2.07)	0.76 (0.3 to 1.91)	.53	
Nonoverexpression	52	55	52			47	40	55		
	1 (referent)	1.11 (0.7 to 1.75)	1.07 (0.54 to 2.1)	.83	.22^b^	1 (referent)	0.84 (0.5 to 1.39)	1.10 (0.51 to 2.39)	.85	.25^b^
Mutation	33	31	28			23	36	29		
	1 (referent)	0.94 (0.5 to 1.77)	0.86 (0.34 to 2.17)	.76		1 (referent)	1.36 (0.7 to 2.66)	0.91 (0.34 to 2.41)	.80	
Wild-type	18	16	21			23	21	35		
	1 (referent)	0.76 (0.31 to 1.86)	0.94 (0.3 to 2.93)	.98	.57^c^	1 (referent)	0.74 (0.35 to 1.55)	0.99 (0.34 to 2.85)	.95	.15^c^
Methionine^g^										
Overall	66	75	110			107	83	63		
	1 (referent)	0.85 (0.56 to 1.3)	1.03 (0.58 to 1.81)	.76		1 (referent)	0.77 (0.51 to 1.15)	0.56 (0.31 to 1.01)	.06	
Overexpression	20	20	49			40	36	27		
	1 (referent)	0.8 (0.37 to 1.71)	1.83 (0.65 to 5.15)	.10		1 (referent)	0.80 (0.42 to 1.52)	0.50 (0.2 to 1.25)	.16	
Nonoverexpression	44	54	61			62	46	34		
	1 (referent)	0.92 (0.55 to 1.54)	0.83 (0.41 to 1.65)	.59	.08^b^	1 (referent)	0.78 (0.46 to 1.33)	0.60 (0.27 to 1.31)	.21	.80^b^
Mutation	24	24	44			33	31	24		
	1 (referent)	0.92 (0.46 to 1.85)	1.84 (0.69 to 4.89)	.13		1 (referent)	0.71 (0.35 to 1.46)	0.42 (0.15 to 1.14)	.09	
Wild-type	19	16	20			32	24	23		
	1 (referent)	0.72 (0.32 to 1.63)	0.86 (0.26 to 2.82)	.75	.08^c^	1 (referent)	0.94 (0.48 to 1.84)	1.18 (0.42 to 3.32)	.78	.50^c^

a Hazard ratio was stratified by study area and adjusted for age, sex, smoking status, body mass index, physical activity, diabetes, dietary intake of calcium and fiber, colorectal cancer screening, vitamin B supplement use, and one–carbon nutrients except for exposure.

b *P* value for heterogeneity between TP53 expression status.

c *P* value for heterogeneity between *TP53* mutation status.

d Tertiles of folate (g/d) were <298.8, 298.8-416.8, and ≥416.8 for men and <338.6, 338.6-458.9, and ≥458.9 for women.

e Tertiles of vitamin B6 (mg/d) were <2.37, 2.37-2.65, and ≥2.65 for men and <2.30, 2.30-2.55, and ≥2.55 for women.

f Tertiles of vitamin B12 (g/d) were <5.83, 5.83-9.28, and ≥9.28 for men and <5.97, 5.97-9.37, and ≥9.37 for women.

g Tertiles of methionine (mg/d) were <1334.7, 1334.8-1624.4, and ≥1624.4 for men and <1277.4, 1277.4-1507.1, and ≥1507.3 for women.

## Discussion

This prospective longitudinal study found the heterogeneous effects of dietary folate and vitamin B6 on CRC risk among subtypes defined by TP53 status. No statistically significant associations with overall CRC and its subtypes were observed for dietary intake of folate, vitamin B12, or methionine. However, vitamin B6 intake was associated with decreased risk of TP53-overexpressing CRC in drinkers and increased risk of *TP53* wild-type CRC in women. Folate intake yielded similar results, with no statistical significance, except in the heterogeneity test. This longitudinal study supports the hypothesis that the effect of one-carbon nutrient intake on CRC differs by tumor condition.

Few studies have examined whether the effect of one-carbon nutrient intake on CRC varies by TP53 status. A report from the Nurses’ Health Study showed that folate and vitamin B6 intakes were inversely associated with the risk of TP53-overexpressing colon cancer,^23^ whereas another report from the Iowa Women’s Health Study suggested no association between folate intake and TP53-overexpressing CRC.^24^ In the present study of dietary intakes of folate, vitamin B6, vitamin B12, and methionine, although the risk of TP53-overexpressing CRC was not reduced in men and women with high intakes of these nutrients, a reduction was observed among drinkers with high vitamin B6 intake. This may be owing to the inhibitory effect of ethanol on the conversion of dietary vitamin B6 into its active coenzyme form, which ultimately leads to vitamin B6 deficiency.^36^ Drinkers with a potential vitamin B6 deficiency might benefit from a high intake of dietary vitamin B6. Meanwhile, the hazard ratio of folate for TP53-overexpressing CRC showed an inverse point estimation in the overall population of this study but did not reach the nominal significance level in any subgroup, including drinkers. In a previous nested case–control study within the JPHC study, only one participant was reported to have folate deficiency (<3 ng/ml), which may have limited our ability to detect an inverse association between folate intake and CRC.^37^ Therefore, the preventive effect of one-carbon nutrient intake on TP53-overexpressing CRC may be more evident in populations with a burden of one-carbon metabolism.

Immunohistochemical TP53 expression in tumors has long been used clinically as a surrogate marker of *TP53* mutations, although it primarily reflects missense mutations with accumulation of aberrant TP53, but not nonsense or other mutations with absent or decreased TP53 expression.^25-27^ In our study, the results for TP53-overexpressing CRC and *TP53*-mutated CRC, including all pathogenic mutations, were similar; however, some differences were observed. For example, an inverse association between vitamin B6 intake and TP53-overexpressing CRC was observed in drinkers, whereas only marginal, non-significant associations were observed for *TP53*-mutated CRC. Similarly, a previous case-control study in which target sequencing was used reported no associations between dietary or supplemental intakes of folate, riboflavin, vitamins B6 and B12, and methionine, and *TP53*-mutated rectal tumor.^38^ Because some *TP53* mutations lead to loss of function while others result in gain-of-function alterations,^39^ it is plausible that all *TP53* mutations should not be combined and that TP53-overexpressing CRC may be a more clinically useful classification.

Nevertheless, interpreting TP53 nonoverexpression is challenging, as this category may include both *TP53* wild-type CRC and those with nonsense mutations, leading to the absence of TP53. To address this, we specifically analyzed *TP53* wild-type CRC based on sequencing results. No associations for TP53-nonoverexpressing CRC were found in any analyses; however, an association between vitamin B6 intake and increased risk of *TP53* wild-type CRC was detected, particularly in women. Folate also appeared to be associated with an increased risk of *TP53* wild-type CRC. Consequently, the heterogeneous effects of vitamin B6 and folate intakes on CRC risk were evident according to *TP53* mutation status, but not TP53 expression status. Interestingly, in the Iowa Women’s Health Study CRC was divided into TP53-negative, TP53-low, and TP53-high groups according to immunohistochemical TP53 expression. Although not statistically significant, folate intake appeared to be positively associated with TP53-low CRC, which is thought to consist largely of *TP53* wild-type CRC.^24^ These findings suggest that the potential protumor effect of one-carbon nutrient intake might be restricted to *TP53* wild-type CRC and that accurate classification of this subtype is essential for evaluating the heterogeneity in the effects of one-carbon nutrient intake on CRC risk. To our knowledge, no other prospective study has examined associations with *TP53* wild-type CRC defined by sequence analysis; further studies are warranted to corroborate the findings of *TP53* wild-type CRC.

Folate and vitamin B6 intakes appeared to be differentially associated with CRC subtypes defined by TP53 status, but not with vitamin B12 and methionine intakes. Vitamin B12 acts as a coenzyme for methionine synthase thereby maintaining methionine levels.^40^ Methionine is then metabolized to S-adenosylmethionine, the primary methyl donor in DNA methylation.^41^ Both nutrients may contribute to the shift to DNA methylation rather than DNA synthesis in one-carbon metabolism. Meanwhile, laboratory studies have revealed that folate and vitamin B6 deficiencies cause severe DNA damage, inducing DNA strand breaks and loss of heterozygosity.^3^^,^^18^^,^^21^^,^^22^ Therefore, a protective effect against CRC subtypes harboring *TP53* mutations may be observed for folate and vitamin B6 intakes. Regarding *TP53* wild-type CRC, in line with our hypothesis that its subtype may share characteristics similar to those of adenomas in which *TP53* is infrequently mutated and the risk is increased by the higher intake of one-carbon nutrients, the statistically significant association of vitamin B6 intake and the marginal association of folate with *TP53* wild-type CRC were found. These findings supported that the temporal increase in CRC incidence in the late 1990s in the United States and Canada was attributable to folate fortification.^13^ However, considering that vitamin B6 is a coenzyme involved in more than 100 metabolic reactions,^42^ these findings may not depend solely on aberrant one-carbon metabolism.

The main strengths of this study are its Prospective Cohort Incident Tumor Biobank Method^43^ and high retrieval rate of tumor tissue. To our knowledge, JPHC is the only Prospective Cohort Incident Tumor Biobank Method–based study in Asia. These features allowed us to assess the associations between long-term exposures and cancer incidence according to tumor characteristics, as well as to estimate cancer incidence rates in a defined population, thereby reducing selection and recall biases inherent in hospital-based case-control designs commonly used in pathological studies. Additionally, we attempted to overcome misclassification biases in exposure and outcome. Dietary intake of one-carbon nutrients was estimated using a validated food frequency questionnaire, and CRC subtypes defined by TP53 status were assessed using immunohistochemistry and DNA sequencing. Nevertheless, we acknowledge some limitations of this study. First, owing to the observational nature of this study, the influence of unknown and unmeasured confounding factors cannot be ruled out. Although a key enzyme in one-carbon metabolism, 5,10-methylenetetrahydrofolate reductase (MTHFR), harbors polymorphisms that alter its activity and shift the balance of one-carbon unit utilization between DNA methylation and DNA synthesis, we could not consider MTHFR polymorphisms because of a lack of genomic information. However, the interaction between one-carbon nutrient intake and MTHFR on CRC has not been confirmed.^44^ Furthermore, adjustment for a wide range of risk factors for CRC and stratification by alcohol intake, which influences the absorption and degradation of vitamin B6 and folate,^42^^,^^45^ minimized the impact of confounding factors. Second, although we used a validated food frequency questionnaire, exposures based solely on self-reported data may have introduced recall bias. Furthermore, approximately 10% of dietary data imputed from the 1995 questionnaire to the 2000 questionnaire may have resulted in misclassification bias. Third, the data were collected more than two decades ago, and dietary habits may have changed during this period. However, we believe the findings remain relevant, as the fundamental biological mechanisms linking one-carbon nutrients to colorectal carcinogenesis have not changed over time. Finally, classification of CRC by TP53 status may have reduced statistical power because of the limited number of cases. Additionally, the cumulative effects of multiple nutrients could not be evaluated. Given the inconsistent directions of associations for one-carbon nutrients across TP53-defined tumor subtypes, further studies are warranted to validate these results.

This prospective longitudinal study demonstrated that the association of folate and vitamin B6 intakes with CRC risk differed by TP53 status. Specifically, vitamin B6 may confer a decreased risk of TP53-overexpressing CRC in drinkers, as well as an increased risk of *TP53* wild-type CRC in women. This longitudinal study provides new insights into the complex mechanisms underlying one-carbon metabolism and CRC risk.

## Supplementary Material

pkag009_Supplementary_Data

## Data Availability

For information on how to submit an application to gain access to JPHC data or biospecimens, please follow the instructions provided at https://epi.ncc.go.jp/en/jphc/805/8155.html. Data supporting the findings of this study are available from the corresponding author upon request.
