# Supplementary material for: Dietary intake of one-carbon nutrients and colorectal cancer risk according to TP53 status
Source: JNCI Cancer Spectr. 2026 Jan 29;10(2):pkag009. doi: 10.1093/jncics/pkag009 (PMC13006204; doi:10.1093/jncics/pkag009)
Supplement: pkag009_Supplementary_Data [file pkag009_supplementary_data.zip › OneCarbon_TP53_SupplementaryMaterial_251224.pdf]

Supplementary Material

Supplementary Methods

Follow-up

Participants in Okinawa were followed up from the date they responded to the questionnaire in 2000, while those in Akita were followed up from January 2004. This is because available tumor tissues were collected from colorectal cancer (CRC) cases diagnosed after 1999 and 2003 in Okinawa and Akita, respectively. From each initiation date to December 31, 2014, the participants' vital and residential statuses were traced annually using the residential registry.

Dietary Assessment

The validity of the estimation of energy-adjusted dietary intake from the food frequency questionnaire was assessed using dietary records of the subgroups in the Japan Public Health Center-based Prospective (JPHC) study. The correlation coefficients for the dietary intakes of folate, vitamin B6, vitamin B12, methionine, calcium, and total fiber were 0.40, 0.45, 0.33, 0.29, 0.54, and 0.41, respectively, for men, whereas the corresponding values were 0.35, 0.47, 0.34, 0.27, 0.45, and 0.41 for women (1, 2). Extreme daily energy values in the highest or lowest 2.5th percentile (<971 or >4377 kcal/day for men and <755 or >3892 kcal/day for women) were treated as missing data.

Supplementary References

1. Ishihara J, Inoue M, Kobayashi M, Tanaka S, Yamamoto S, Iso H, et al. Impact of the revision of a nutrient database on the validity of a self-administered food frequency questionnaire (FFQ). J Epidemiol. 2006;16(3):107-16.
2. Okada C, Iso H, Ishihara J, Maruyama K, Sawada N, Tsugane S, et al. Validity and reliability of a self-administered food frequency questionnaire for the JPHC study: The assessment of amino acid intake. J Epidemiol. 2017;27(5):242-7.

Table S1. Baseline characteristics of participants according to dietary intake of vitamin B12 and methionine

		Vitamin B12				Methionine			
		Q1	Q2	Q3	Q4	Q1	Q2	Q3	Q4
Characteristics		N=5029	N=5070	N=5094	N=5061	N=5055	N=5100	N=5080	N=5019
Age		60.0 (6.1)	60.9 (6.2)	61.9 (6.2)	63.1 (6.1)	60.3 (6.2)	61.2 (6.2)	61.7 (6.3)	62.6 (6.3)
Men, N (%)		2346 (46.6)	2360 (46.5)	2366 (46.4)	2351 (46.5)	2360 (46.7)	2378 (46.6)	2363 (46.5)	2322 (46.3)
BMI, Mean (SD)		24.4 (3.3)	24.1 (3.2)	23.8 (3.1)	23.6 (3.0)	24.1 (3.3)	24.0 (3.1)	23.9 (3.1)	23.9 (3.2)
METs, Mean (SD)		33.5 (5.8)	33.5 (5.6)	33.5 (5.7)	33.3 (5.7)	33.7 (5.8)	33.5 (5.6)	33.3 (5.6)	33.2 (5.8)
Diabetes, N (%)		240 (4.8)	247 (4.9)	260 (5.1)	336 (6.6)	191 (3.8)	249 (4.9)	291 (5.7)	352 (7.0)
Colorectal screening, N (%)		397 (7.9)	454 (9.0)	544 (10.7)	563 (11.1)	413 (8.2)	470 (9.2)	551 (10.8)	524 (10.4)
Smoking, N (%)	Never	3319 (66.0)	3349 (66.1)	3327 (65.3)	3269 (64.6)	3139 (62.1)	3338 (65.5)	3385 (66.6)	3402 (67.8)
	Past	610 (12.1)	688 (13.6)	661 (13.0)	687 (13.6)	610 (12.1)	677 (13.3)	692 (13.6)	667 (13.3)
	Current	1100 (21.9)	1033 (20.4)	1106 (21.7)	1105 (21.8)	1306 (25.8)	1085 (21.3)	1003 (19.7)	950 (18.9)
Alcohol drinking,	Non	3367 (67.0)	3217 (63.5)	3037 (59.6)	2826 (55.8)	2779 (55.0)	3118 (61.1)	3269 (64.4)	3281 (65.4)
N (%)	Occasional	446 (8.9)	585 (11.5)	679 (13.3)	786 (15.5)	404 (8.0)	616 (12.1)	706 (13.9)	770 (15.3)
	Light	405 (8.1)	481 (9.5)	523 (10.3)	607 (12.0)	458 (9.1)	559 (11.0)	499 (9.8)	500 (10.0)
	Heavy	811 (16.1)	787 (15.5)	855 (16.8)	842 (16.6)	1414 (28.0)	807 (15.8)	606 (11.9)	468 (9.3)
Vitamin B supplement use, N (%)		460 (9.1)	559 (11.0)	638 (12.5)	635 (12.5)	472 (9.3)	569 (11.2)	623 (12.3)	628 (12.5)
Dietary intake, Median (IQR)[a]									
Energy (kcal/d)		1845.4	1838.6	1926.1	1864.6	1884.5	1857.8	1864.9	1867.4
		(1414.7–2377.8)	(1461.8–2335)	(1539.6–2420.8)	(1482.3–2344.6)	(1447.9–2395)	(1463.4–2367.7)	(1494.6–2344.6)	(1484.8–2369.6)
Calcium (mg/d)		396.0	468.1	496.0	517.8	386.7	454.1	511.9	544.5
		(286–533.6)	(348.2–614)	(383.5–626.9)	(404.1–659.6)	(285.2–505.5)	(345.1–586.1)	(392.6–646.9)	(416.1–714.6)
Fiber (g/d)		12.7	13.5	14.3	15.0	13.1	13.8	14.3	14.1
		(9.4–16.4)	(10.3–17)	(11.3–17.8)	(11.9–18.4)	(9.5–17.1)	(10.8–17.5)	(11.3–17.7)	(11.1–17.5)
Folate (g/d)		332.8	358.9	388.6	418.4	343.6	370.8	389.7	399.8

	(243.3–451.3)	(279.8–462.8)	(307.8–483.8)	(338.8–510.1)	(251.7–460.9)	(287.4–473.6)	(305.8–486)	(320.1–496.7)
Vitamin B6 (mg/d)	2.2 (2.1–2.4)	2.4 (2.2–2.5)	2.5 (2.4–2.7)	2.7 (2.5–2.9)	2.3 (2.1–2.5)	2.4 (2.2–2.6)	2.5 (2.3–2.7)	2.7 (2.5–2.9)
Vitamin B12 (g/d)	4.0 (3.2–4.6)	6.3 (5.7–6.9)	8.8 (8.1–9.6)	13.2 (11.7–15.6)	4.6 (3.4–6.1)	6.6 (5.1–8.4)	8.5 (6.7–10.7)	11.9 (9.2–15)
Methionine (mg/d)	1174.8 (1028.9–1323.7)	1347.7 (1219.5–1490.6)	1484.0 (1349.5–1633.9)	1713.0 (1544.7–1908.7)	1106.1 (997.4–1176.5)	1331.8 (1285.7–1378.3)	1522.7 (1469.7–1579.5)	1818.5 (1717.8–1973.4)

BMI, body mass index (kg/m^2); METs, Metabolic Equivalent for Tasks (hours/day); IQR, interquartile range.

[a] The intake was adjusted for energy intake using a residual method.

Table S2. Crude hazard ratios of p53–defined colorectal cancer according to one–carbon nutrients

	Overall		Immunohistochemistry				DNA sequence			
			Overexpression		Non–overexpression		Mutation		Wild–type	
	N	HR (95%CI)	N	HR (95%CI)	N	HR (95%CI)	N	HR (95%CI)	N	HR (95%CI)
Folate										
Q1	113	1 (reference)	43	1 (reference)	68	1 (reference)	39	1 (reference)	30	1 (reference)
Q2	129	1.06 (0.82 – 1.37)	52	1.13 (0.75 – 1.7)	74	1.01 (0.73 – 1.41)	56	1.38 (0.91 – 2.09)	26	0.78 (0.46 – 1.32)
Q3	135	1.06 (0.83 – 1.37)	50	1.05 (0.69 – 1.58)	82	1.07 (0.78 – 1.48)	44	1.05 (0.68 – 1.63)	39	1.11 (0.69 – 1.8)
Q4	127	0.97 (0.75 – 1.26)	47	0.95 (0.62 – 1.46)	77	0.99 (0.71 – 1.37)	41	0.94 (0.6 – 1.47)	39	1.13 (0.69 – 1.83)
Ptrend		0.84		0.72		0.97		0.45		0.35
Vitamin B6										
Q1	110	1 (reference)	36	1 (reference)	73	1 (reference)	37	1 (reference)	24	1 (reference)
Q2	114	0.95 (0.73 – 1.23)	47	1.22 (0.78 – 1.89)	62	0.77 (0.55 – 1.08)	46	1.19 (0.77 – 1.85)	31	1.13 (0.66 – 1.91)
Q3	142	1.14 (0.88 – 1.48)	56	1.42 (0.92 – 2.21)	84	1.01 (0.73 – 1.39)	53	1.37 (0.89 – 2.13)	30	1.02 (0.6 – 1.74)
Q4	138	1.07 (0.82 – 1.39)	53	1.32 (0.84 – 2.07)	82	0.94 (0.69 – 1.3)	44	1.14 (0.71 – 1.81)	49	1.57 (0.97 – 2.54)
Ptrend		0.34		0.18		0.82		0.47		0.08
Vitamin B12										
Q1	115	1 (reference)	41	1 (reference)	72	1 (reference)	45	1 (reference)	29	1 (reference)
Q2	121	1.02 (0.78 – 1.32)	39	0.96 (0.61 – 1.51)	79	1.04 (0.75 – 1.43)	44	1.01 (0.66 – 1.54)	29	0.86 (0.51 – 1.44)
Q3	130	1.04 (0.79 – 1.35)	57	1.39 (0.9 – 2.15)	70	0.86 (0.61 – 1.21)	47	1.09 (0.69 – 1.71)	27	0.69 (0.4 – 1.2)
Q4	138	1.07 (0.81 – 1.42)	55	1.35 (0.84 – 2.18)	80	0.94 (0.66 – 1.34)	44	1.04 (0.63 – 1.72)	49	1.16 (0.7 – 1.91)
Ptrend		0.61		0.10		0.54		0.78		0.54
Methionine										
Q1	135	1 (reference)	45	1 (reference)	85	1 (reference)	45	1 (reference)	42	1 (reference)
Q2	111	0.76 (0.59 – 0.98)	42	0.87 (0.57 – 1.33)	66	0.72 (0.52 – 0.99)	37	0.78 (0.5 – 1.2)	32	0.69 (0.43 – 1.09)
Q3	112	0.76 (0.59 – 0.98)	42	0.87 (0.57 – 1.33)	69	0.74 (0.54 – 1.02)	44	0.93 (0.61 – 1.42)	20	0.42 (0.24 – 0.71)
Q4	146	0.97 (0.76 – 1.23)	63	1.29 (0.86 – 1.92)	81	0.85 (0.62 – 1.16)	54	1.15 (0.76 – 1.73)	40	0.81 (0.52 – 1.25)
Ptrend		0.91		0.21		0.41		0.37		0.22

HR:hazard ratio. HR was stratified by study area and adjusted for age and sex.

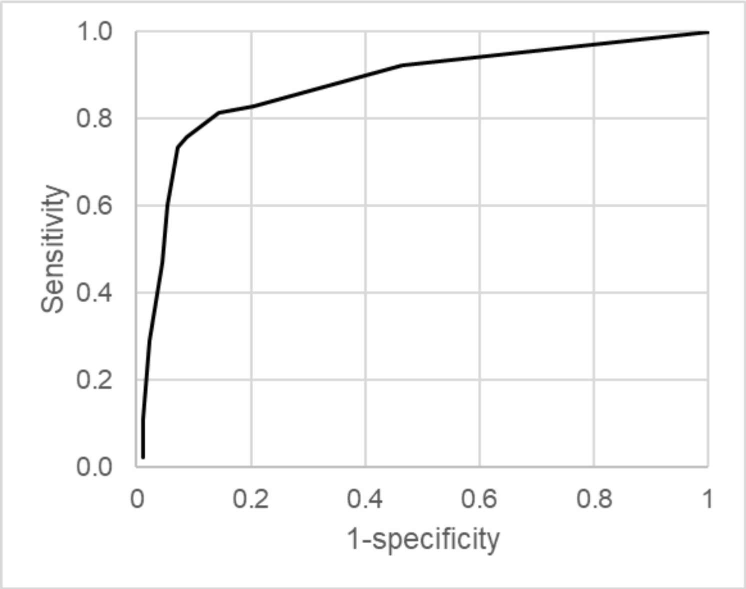

Figure S1. Receiver operating characteristic curves of prediction of *TP53* missense mutation using p53 expression
